# Adaptation of Mitochondrial Substrate Flux in a Mouse Model of Nonalcoholic Fatty Liver Disease

**DOI:** 10.3390/ijms21031101

**Published:** 2020-02-07

**Authors:** Pavla Staňková, Otto Kučera, Eva Peterová, Halka Lotková, Tumisang Edward Maseko, Kateřina Nožičková, Zuzana Červinková

**Affiliations:** 1 Department of Physiology, Faculty of Medicine in Hradec Králové, Charles University, Šimkova 870, 500 03 Hradec Králové, Czech Republic; stankovap@lfhk.cuni.cz (P.S.); lotko@lfhk.cuni.cz (H.L.); MASEKOT@lfhk.cuni.cz (T.E.M.); katerina.nozickova@fnhk.cz (K.N.); wolff@lfhk.cuni.cz (Z.Č.); 2 Department of Medical Biochemistry, Faculty of Medicine in Hradec Králové, Charles University, Šimkova 870, 500 03 Hradec Králové, Czech Republic; PeterovaE@lfhk.cuni.cz

**Keywords:** nonalcoholic fatty liver disease, mitochondria, oxidative phosphorylation, respirometry

## Abstract

Maladaptation of mitochondrial oxidative flux seems to be a considerable feature of nonalcoholic fatty liver disease (NAFLD). The aim of this work was to induce NAFLD in mice fed a Western-style diet (WD) and to evaluate liver mitochondrial functions. Experiments were performed on male C57BL/6J mice fed with a control diet or a WD for 24 weeks. Histological changes in liver and adipose tissue as well as hepatic expression of fibrotic and inflammatory genes and proteins were evaluated. The mitochondrial respiration was assessed by high-resolution respirometry. Oxidative stress was evaluated by measuring lipoperoxidation, glutathione, and reactive oxygen species level. Feeding mice a WD induced adipose tissue inflammation and massive liver steatosis accompanied by mild inflammation and fibrosis. We found decreased succinate-activated mitochondrial respiration and decreased succinate dehydrogenase (SDH) activity in the mice fed a WD. The oxidative flux with other substrates was not affected. We observed increased ketogenic capacity, but no impact on the capacity for fatty acid oxidation. We did not confirm the presence of oxidative stress. Mitochondria in this stage of the disease are adapted to increased substrate flux. However, inhibition of SDH can lead to the accumulation of succinate, an important signaling molecule associated with inflammation, fibrosis, and carcinogenesis.

## 1. Introduction

The liver is a principal metabolic organ that governs body energy metabolism. Nonalcoholic fatty liver disease (NAFLD) is currently the most common chronic liver condition worldwide [1]. In the vast majority of cases, it is the consequence of chronic overnutrition, and NAFLD is now recognized as a hepatic manifestation of metabolic syndrome [2]. NAFLD encompasses a wide range of pathologies, including simple steatosis, nonalcoholic steatohepatitis (NASH), fibrosis, and ultimately cirrhosis, which may progress to hepatocellular carcinoma. The mechanism for the development and progression of NAFLD is multifactorial, and there are still substantial gaps in knowledge regarding NAFLD pathogenesis and treatment [3,4]. NAFLD is not simply a liver pathology; it is accompanied by whole-body metabolic changes.

At the systemic level, lipids are at the bottom of an oxidative hierarchy, and their utilization depends on the presence of other fuels [5]. The ability to freely switch between alternative fuels according to physiological and nutritional circumstances is called metabolic flexibility [6], and it relies on the action of insulin, glucagon, and other hormones. In the fed state, the insulin action removes competition for substrate utilization. When the storage capacity of the subcutaneous adipose tissue (AT) is exceeded, or impaired, insulin is not able to inhibit AT lipolysis, and fat begins to accumulate in other organs [7]. Insulin-resistant individuals continue to oxidize a mixture of fuels regardless of the nutritional context [6].

Oxidation of fatty acids (FAs) and other substrates occurs mainly in the mitochondria. Mitochondrial dysfunction and oxidative stress are detected in liver tissues from NAFLD patients [8]. However, studies of mitochondrial function in NAFLD are conflicting. The broad spectrum of conditions covered by the term NAFLD makes it difficult to compare studies directly, and alterations in mitochondrial energetics depend on the stage of the diseases, the susceptibility of the metabolic pathway, and the ability of hepatocytes to buffer and store excess lipids [9,10]. A better understanding of the mitochondrial pathways and their adaptive/maladaptive responses in NASH is essential. Without understanding the respiratory activities of mitochondria, it is impossible to understand other processes in mitochondria and cells [11].

Total caloric intake represents a key regulator of liver fat content [12]. It also appears that the contribution of nutrients and/or a combination of nutrients to the development and progression of NAFLD varies [12,13,14]. Fructose is metabolized preferentially in the liver and provides a source of energy that is not regulated by the energy status of the cell. High amounts of saturated fat, glucose, and/or fructose is a typical feature of the Western-style diet (WD) [12,15,16]. The development of obesity and NAFLD in humans occurs over years, and there are limitations to human tissue acquisition. Animal models that mimic human pathology are thus necessary. The WD-fed animal models most closely resemble the human condition [2,3,17,18]. The definition of a WD can differ based on composition and the percentage and types of fat, cholesterol, and sugar included [19]. The severity of diet-induced NAFLD also depends on the species, gender, animal strain, and substrain [16,20]. Reproducibility is of crucial importance in any animal model of disease. For this reason, we selected the WD mouse model of NASH using a standard commercially available diet and mice strain [18]. As changes in energy metabolism depend on the stage of the disease, a longitudinal dynamic analysis of NAFLD progression in this model was performed by the same research group [21].

The aim of this work was to reproduce a model of fibrosis-developing NASH in C57BL/6J mice fed a high-fat, high-fructose, and high-cholesterol WD [18,21]. We selected a feeding interval of 24 weeks, when noticeable liver inflammation and fibrosis were observed [21]. Because in vivo different available substrates simultaneously feed electrons to the electron transfer system (ETS), notably in the state of metabolic inflexibility, we evaluated liver mitochondrial functions using a mixture of respiratory substrates and substrate-uncoupler-inhibitor titration reference protocols [22].

## 2. Results

### 2.1. Whole Body, Liver, and Epididymal Fat Weight

Animals fed the WD gained significantly more weight (*p* < 0.01), and their absolute and relative liver weights and the amount of epididymal fat were higher in comparison with the mice fed the control diet (CD) (*p* < 0.01) (Table 1).

### 2.2. Blood Analysis

The activity of alanine transaminase (ALT) was higher in the mice fed the WD (*p* < 0.01), as were the cholesterol and bile acid concentrations (*p* < 0.01). The level of blood urea nitrogen (BUN) was lower in comparison with that of the control animals (*p* < 0.01). The activities of γ-glutamyl transferase (GGT) and alkaline phosphatase (ALP), as well as the concentrations of albumin and total bilirubin, were not significantly affected (Table 1).

### 2.3. Epididymal Fat (eWAT)

**Figure 1:**
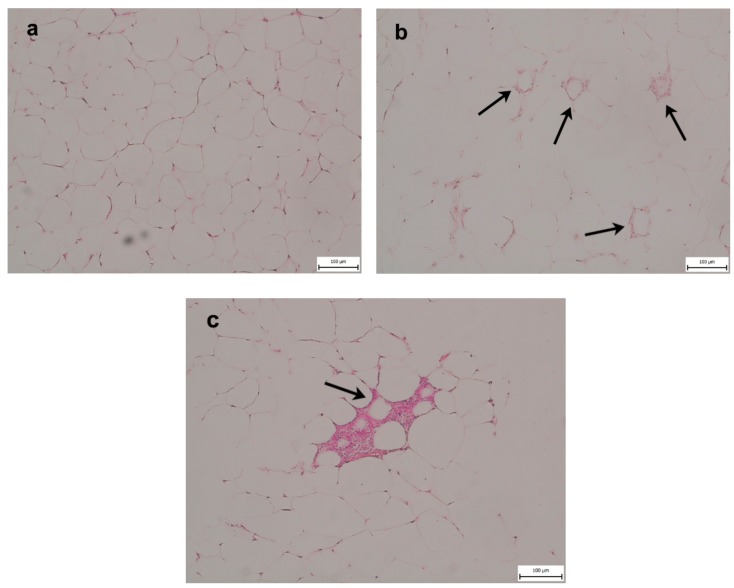
Epididymal fat histology (**a**) in mice fed a control diet (CD) and (**b**,**c**) Western-style diet (WD). Macrophage infiltration showing formation of crown-like structures (indicated by arrows) characteristic of stressed insulin-resistant dying adipocytes in the WD group. No indications of inflammation are seen in the CD group.

We observed characteristic macrophage crown-like structures surrounding dying or dead adipocytes in the eWAT of all mice reared on WD. Mice fed the CD showed no evidence of eWAT inflammation (Figure 1).

### 2.4. Steatosis, Inflammation, Fibrosis, and Apoptosis

**Figure 2:**
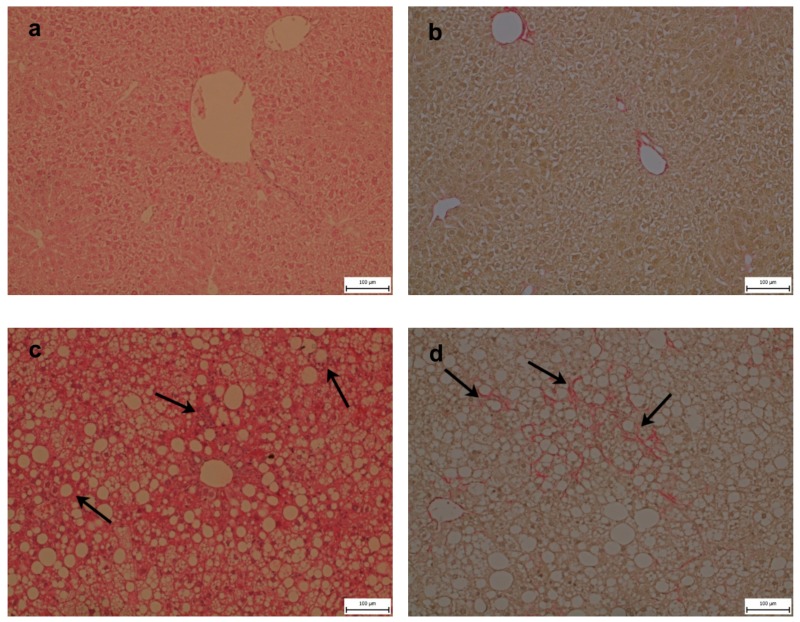
Liver histology (**a**,**b**) in mice fed a control diet (CD) and (**c**,**d**) Western-style diet (WD). (**a**,**c**) Hematoxylin and eosin sections of mice fed a WD reveal massive mixed steatosis with inflammatory infiltrated cells indicated by arrows. (**b**,**d**) Sirius red staining revealed mild perisinusoidal fibrosis indicated by arrows.

**Figure 3:**
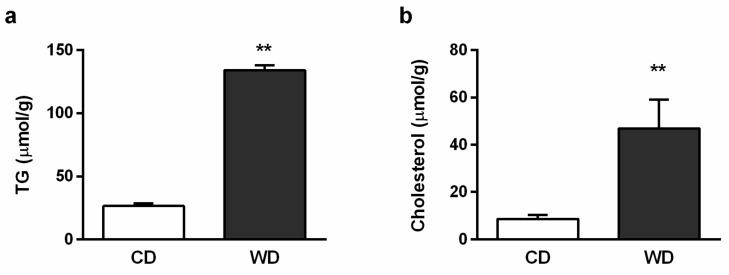
Hepatic lipids in mice fed a control diet (CD) and a Western-style diet (WD). (**a**) Total triglycerides (TGs) and (**b**) cholesterol content. The results are expressed as the means ± SD; ** *p* < 0.01 (*n* = 6 each group).

**Figure 4:**
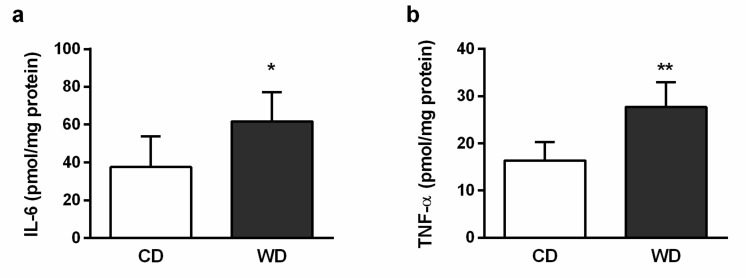
Hepatic inflammatory cytokines in mice fed a control diet (CD) and a Western-style diet (WD). (**a**) Levels of liver interleukin 6 (IL-6) and (**b**) tumor necrosis factor alpha (TNF-α). The results are expressed as the means ± SD; * *p* < 0.05, ** *p* < 0.01 (*n* = 6 each group).

**Figure 5:**
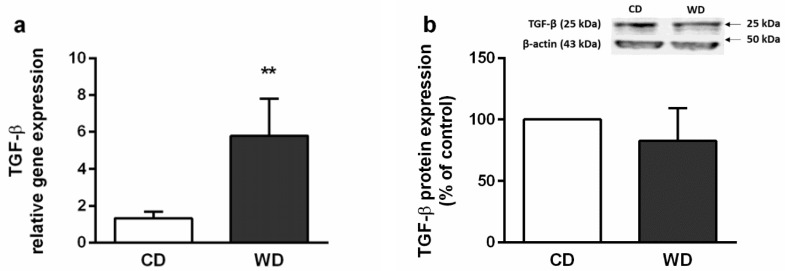

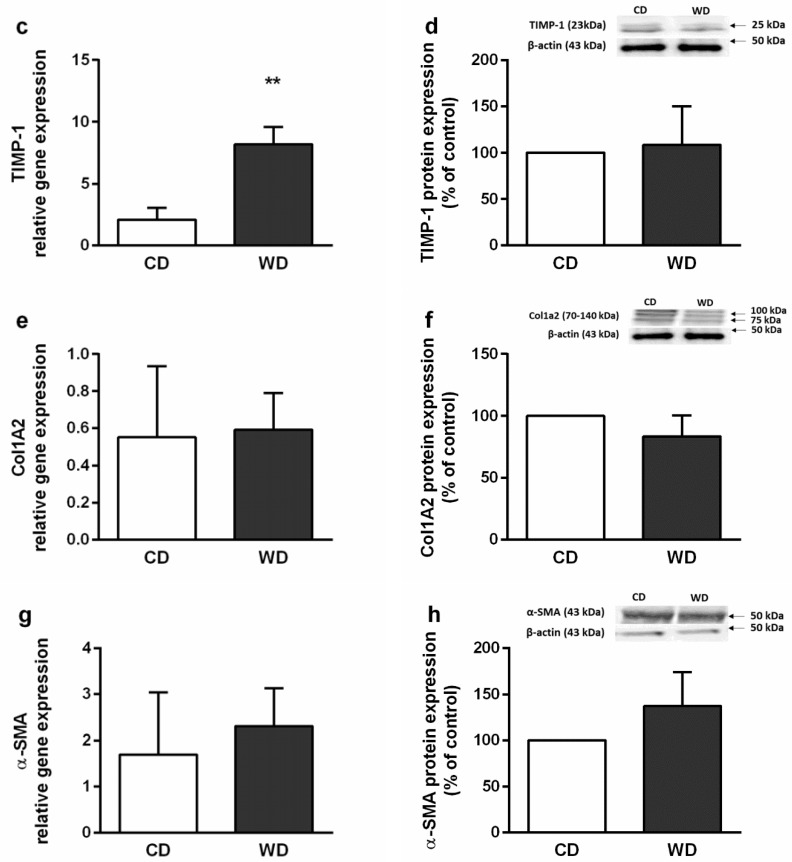
Markers of hepatic fibrosis and stellate cell activation in mice fed a control diet (CD) and a Western-style diet (WD). (**a**) Tumor growth factor beta (TGF-β) gene and (**b**) TGF-β protein expression. (**c**) Tissue inhibitor of metalloproteinase-1 (TIMP-1) gene and (**d**) TIMP-1 protein expression. (**e**) Collagen type I alpha 2 chain (Col1A2) gene and (**f**) Col1A2 protein expression. (**g**) Alpha smooth muscle actin (α-SMA) gene and (**h**) α-SMA protein expression. The results are expressed as the means ± SD; ** *p* < 0.01 (*n* = 4–5 each group). Representative Western blot images are shown in graphs.

**Figure 6:**
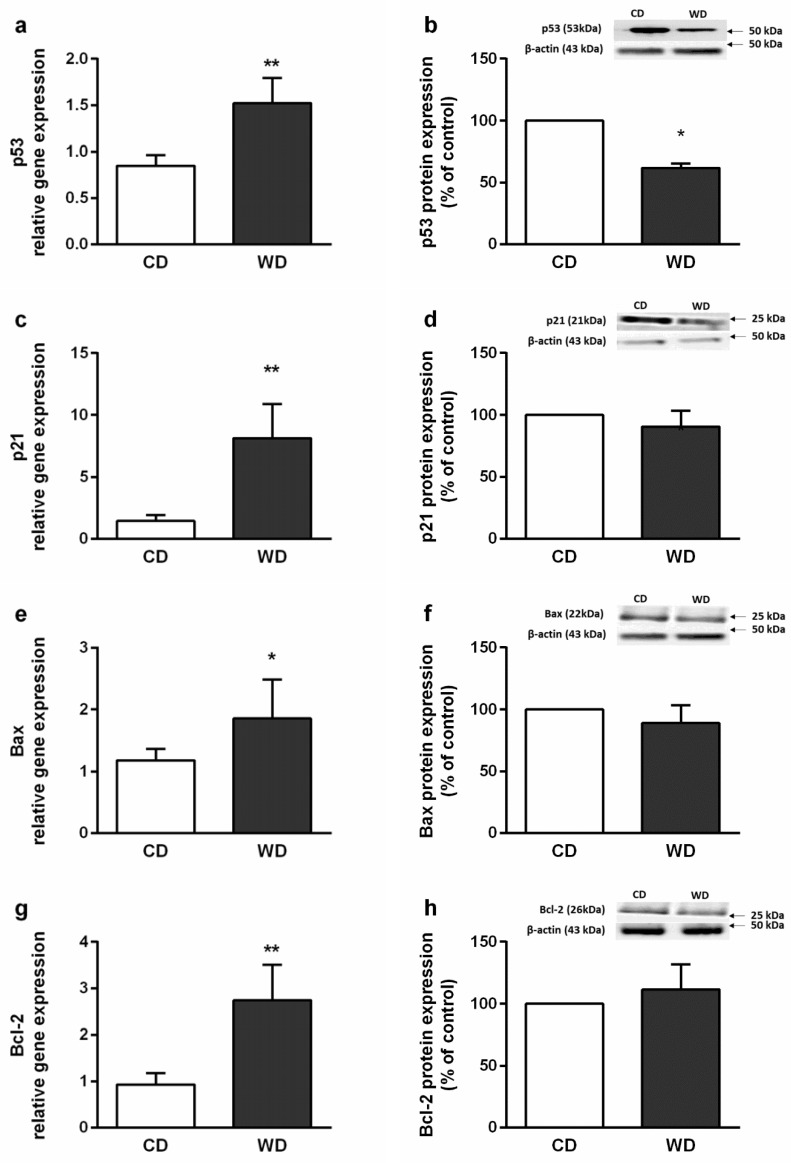
Markers of hepatic apoptosis in mice fed a control diet (CD) and a Western-style diet (WD). (**a**) Transcription factor p53 gene and (**b**) p53 protein expression. (**c**) Cyclin-dependent kinase inhibitor 1 (p21) gene and (**d**) p21 protein expression. (**e**) Proapoptotic protein Bax gene and (**f**) Bax protein expression. (**g**) Antiapoptotic protein Bcl-2 gene and (**h**) Bcl-2 protein expression. The results are expressed as the means ± SD; * *p* < 0.05, ** *p* < 0.01 (*n* = 4–5 each group). Representative Western blot images are shown in graphs.

Histologically, the severity of the steatosis, inflammation, and fibrosis was evaluated using the NASH Clinical Research Network Scoring System [23]. All six animals in the WD group received a maximum score of 3 for steatosis. Steatosis was present in more than 98% of the hepatocytes and was found in both macrovesicular (60%) and microvesicular (40%) forms. Mild inflammation (score 1) was observed in three animals, and mild perisinusoidal fibrosis (score 1A) was observed in four animals from the WD group. There was no evidence of steatosis and fibrosis in the control group, and one mouse from this group received a score of 1 for inflammation. Hepatocellular ballooning was not observed (Figure 2). Steatosis in the WD group was confirmed by the increased level of the tissue triglycerides (TGs) and cholesterol (Figure 3), and mild inflammation by the level of liver tumor necrosis factor alpha (TNF-α) and interleukin 6 (IL-6) (Figure 4). Although we observed a significant (*p* < 0.01) increase in hepatic gene expression of the regulators of fibrosis, transforming growth factor beta (TGF-β), and tissue inhibitor of metalloproteinases-1 (TIMP-1), in the WD group, we did not confirm their increased expression at the protein level (Figure 5 a–d). The gene and protein expression levels of collagen type I alpha 2 chain (Col1A2) and alpha smooth muscle actin (α-SMA) were not significantly affected by the diet intake (Figure 5 e–h). Gene expression of transcription factor p53 and cyclin-dependent kinase inhibitor 1 (p21) and the apoptotic proteins Bax and Bcl-2 were significantly higher in the WD group, but this increase was not confirmed at the protein level (Figure 6).

### 2.5. Oxidative Stress

**Figure 7:**
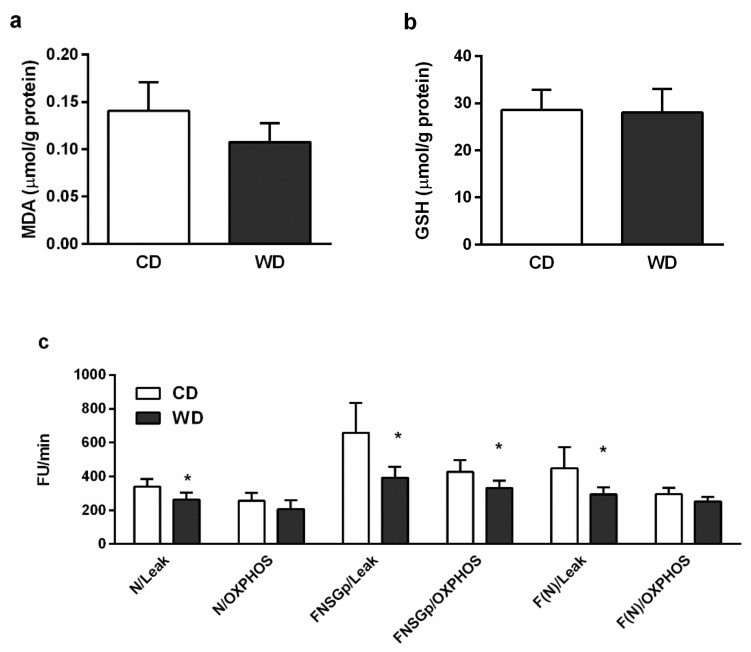
Markers of hepatic oxidative stress in mice fed a control diet (CD) and a Western-style diet (WD). (**a**) Concentration of hepatic malondialdehyde (MDA). (**b**) Concentration of hepatic reduced glutathione (GSH). (**c**) Reactive oxygen species (ROS) production in liver homogenate measured as fluorescence changes in dichlorofluorescein. LEAK, measurements in the absence of adenosine diphosphate (ADP); oxidative phosphorylation (OXPHOS), measurements in the presence of ADP (2.5 mM); N, NADH-linked substrates (pyruvate, 5 mM; glutamate, 10 mM; malate, 2 mM); FNSGp, fatty acid (octanoylcarnitine, 0.5 mM) plus NADH-linked substrates plus succinate (10 mM) and glycerophosphate (10 mM); F(N), octanoylcarnitine (0.5 mM) plus malate (2 mM). The results are expressed as the means ± SD; * *p* < 0.05 (*n* = 6 each group).

**Figure 8:**
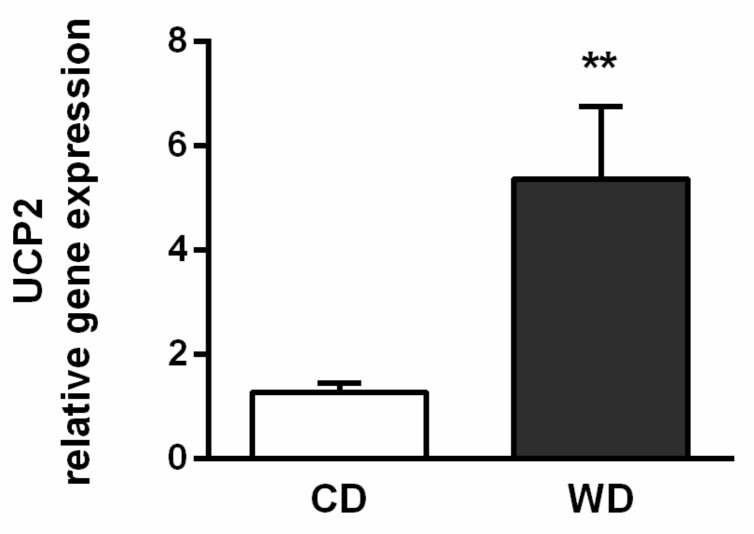
Uncoupling protein 2 (UCP-2) gene expression in mice fed a control diet (CD) and a Western-style diet (WD). The results are expressed as the means ± SD; ** *p* < 0.01 (*n* = 4 each group).

We did not confirm the presence of oxidative stress. Reduced glutathione (GSH) levels were equivalent in the liver homogenates from mice fed the CD and WD diets (Figure 7b), and there was no significant difference in hepatic concentrations of thiobarbituric acid reactive substances (TBARS) (Figure 7a). To measure reactive oxygen species (ROS) production, the liver homogenates were incubated with a mixture of NADH-linked substrates, a mixture of NADH- and FADH_2_-linked substrates, and/or octanoylcarnitine in the presence (oxidative phosphorylation (OXPHOS) state) or absence of adenosine diphosphate (ADP) (leak state). At high redox potential (leak state), the production of ROS was significantly lower (*p* < 0.05) in the WD group with all the experimental substrate mixtures and also with NADH plus FADH_2_-linked substrates in the OXPHOS state in comparison with the CD group (Figure 7c). We found increased gene expression of uncoupling protein 2 (UCP-2) (Figure 8) in the mice fed the WD (*p* < 0.01).

### 2.6. Mitochondrial Respiration

**Figure 9:**
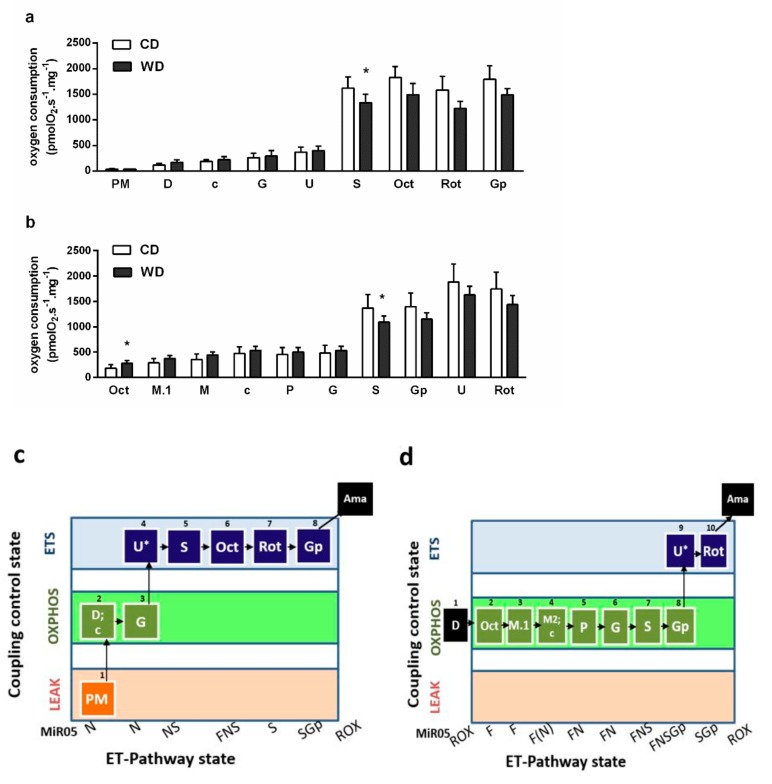
Mitochondrial respiration in mice fed a control diet (CD) and a Western-style diet (WD). (**a**) Respiratory protocol 1 (RP1). (**b**) Respiratory protocol 2 (RP2). (**c**) Coupling/pathway control diagram RP1. (**d**) Coupling/pathway control diagram RP2. Experiments were performed in 2 mL of mitochondrial respiratory medium MiR05. Liver homogenates were loaded at a protein concentration of 0.2 mg/mL, and substrates, uncoupler, and inhibitors were gradually added according to the protocol: P (pyruvate, 5 mM), M (malate, 2 mM), M.1 (malate, 0.1 mM), D (ADP, 2.5 mM), c (cytochrome c, 10 µM), G (glutamate, 10 mM), U (carbonyl cyanide 4-(trifluoromethoxy)phenylhydrazone, 1.5–2 µM), S (succinate, 50 mM), Oct (octanoylcarnitine, 0.5 mM), Rot (rotenone, 0.5 µM), and Gp (glycerophosphate, 10 mM). Finally, antimycin A (2.5 µM) was introduced, and the data were corrected for residual oxygen consumption (ROX) as the baseline state. The results are expressed as the means ± SD; * *p* < 0.05 (*n* = 6 each group). The LEAK state was measured in the presence of reducing substrates, but the absence of ADP, representing electron flow coupled to proton pumping to compensate for proton leaks. Oxidative phosphorylation (OXPHOS) capacity was measured in the presence of saturating concentrations of ADP (D) and defined reduced substrates. Electron transfer (ET) system (ETS) capacity was measured as oxygen consumption in the noncoupled state at optimum uncoupler (U) concentration, which was obtained by stepwise titration (*) to induce maximum oxygen flux. The NADH ET-pathway state (N) was obtained by the addition of NADH-linked substrates (P, M, G). Succinate-induced respiratory state (S) supported the electron flux through complex II. Fatty acid (Oct) oxidation pathway control state (F) feeds electrons through fatty acyl-CoA dehydrogenase to an electron transfer flavoprotein and further to ubiquinone. Glycerophosphate dehydrogenase complex oxidizes Gp and feeds electrons directly to ubiquinone. Residual oxygen consumption (ROX) is respiration due to oxidative side reactions that continue after inhibition of the ET-pathway (Rot, inhibitor of respiratory complex I; Ama, inhibitor of complex III) or in mitochondrial preparations incubated without the addition of fuel substrates. Outer mitochondrial membrane integrity was assessed by the addition of cytochrome c (c).

**Figure 10:**
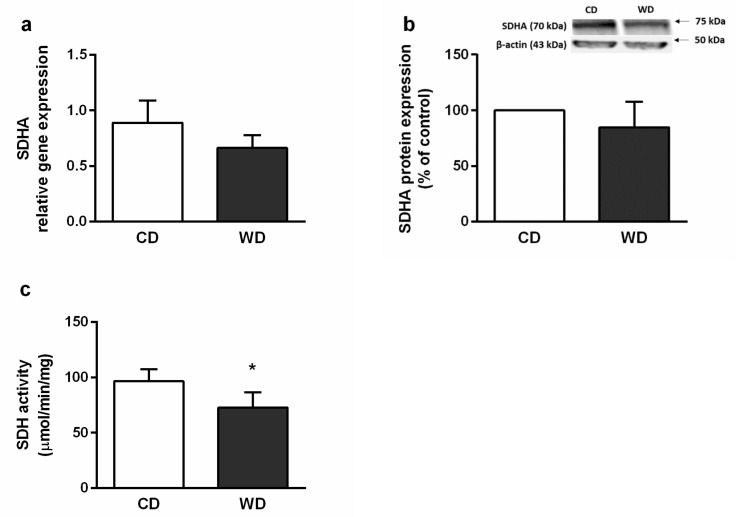
Hepatic succinate dehydrogenase (SDH) in mice fed a control diet (CD) and a Western-style diet (WD). (**a**) SDH subunit A (SDHA) relative gene expression. (**b**) SDH subunit A protein expression. (**c**) SDH enzymatic activity. The results are expressed as the means ± SD; * *p* < 0.05 (*n* = 4–6 each group).

We observed lower oxygen consumption after the addition of succinate in both the OXPHOS and electron transfer (ET) states (*p* < 0.05) and higher oxygen consumption induced by octanoylcarnitine in the absence of malate (*p* < 0.05), which reflects liver ketogenesis [24] (Figure 9). We also confirmed decreased enzymatic activity of SDH (*p* < 0.05) (Figure 10c). We did not find significant differences in the gene or protein expression levels of subunit A of SDH (SDHA) (Figure 10a,b).

## 3. Discussion

Obesity and chronic low-grade inflammation are important risk factors for the onset of NAFLD. The weight of the animals fed the WD (45.17 ± 4.02 g) was significantly higher compared with that of the controls (30.5 ± 3.27 g). In male C57BL/6J mice fed a high-fat diet (HFD), a body weight of approximately 40 g was estimated as a critical tipping point beyond which metabolic dysfunction occurred. At this body weight, eWAT adipocytes were saturated and could not grow to store additional fat [25]. Accordingly, we found more eWAT in mice fed the WD with inflammatory cell infiltration and the presence of crown-like structures compared with the control mice, which did not exhibit marks of eWAT inflammation. The inability to safely store excess energy in the form of TGs in AT is connected to insulin resistance and metabolic inflexibility [7].

The blood analysis revealed mild hepatic injury in mice fed the WD. The liver fat content was dramatically increased in these animals, and we observed massive mixed steatosis. However, liver TGs content per se is not always predictive of NASH progression [3,26]. Increasing evidence indicates that TGs’ deposition in nonadipose cells provides a mechanism by which an organism handles excess FAs and diverts them from various cytotoxic pathways [9,27,28].

Although we found elevated liver enzymes in the blood of the mice in the WD group, we did not observe hepatocellular ballooning. Liver inflammation and fibrosis in mice fed the WD were milder in comparison with the mice in the studies of Charlton et al. [18] and Krishnan et al. [21]. However, there were also differences in liver injury between the specimens in these two studies. In the former study, ballooning was present after 25 weeks of WD feeding; in the latter study, no ballooning was detected despite a prolonged feeding interval (36 weeks). A possible explanation for the discrepancy could be related to animal housing variables. In the Charlton et al. study, the mice were maintained in individual cages; in the Krishnan et al. longitudinal study, the mice were housed two mice to a cage; and in our study, the mice were housed six to a cage. Social isolation of animals has been shown to induce changes in corticosterone levels, indicating an immuno-response, and affects reactivity to a stressor [29,30]. Increasing the group size of mice was associated with decreased food intake and increased level of physical activity [31]. These differences in maintenance could partly explain the differences among these studies and highlight the importance of animal housing conditions as one of the crucial factors affecting metabolic responses.

Competition between substrates and excessive mixed nutrient entry into the ETS not matched by energy demand may overload the ETS, resulting in ROS production [32]. Oxidative stress is thought to play a significant role in NAFLD and NASH progression, although a cause-and-effect type of relationship has not yet been established [33,34]. Considering the level of GSH and malondialdehyde (MDA), we did not observe a difference in oxidative stress in the mice fed the WD in comparison with control animals. This finding is consistent with those of Charlton et al. [18] and Krishnan et al. [21], who did not find increased levels of 8-hydroxydeoxyguanosine or 4-hydroxynonenal, respectively. These results could be affected by redox alterations in C57BL/6J mice owing to a mutation in the nicotinamide nucleotide transhydrogenase enzyme [20]. However, in a human study, elevated oxidative stress was detected only in patients with histological evidence of NASH, but not in patients with simple steatosis [35]. We also measured ROS production in the liver homogenates incubated with a mixture of NADH-linked substrates, a mixture of NADH- and FADH_2_-linked substrates, and/or octanoylcarnitine in the presence or absence of ADP. In the absence of ADP, which mimics the state of low-energy demand, the production of ROS was significantly lower in the WD group with all the substrate mixtures examined. With the NADH- and FADH_2_-linked substrates, the ROS production was lower, even in the presence of ADP, in comparison with the controls. Unfortunately, we have not estimated the protein expression and the effect of UCP-2 on ROS production must be elaborated on by further studies.

We performed a detailed analysis of mitochondrial respiration. The respiration using NADH-dependent substrates was similar in both groups. In the WD group, we observed decreased succinate-activated respiration in the presence of NADH-dependent substrates in both the ET and OXPHOS states. We also found decreased SDH activity. This finding is in agreement with other studies. Decreased maximal ET capacity and succinate-activated ET capacity without affecting NADH-dependent respiration were observed in the livers of the obese (db/db) mice [36]. Decreased activity of liver SDH and increased plasmatic succinate concentration were found in the C57BL/6J mice fed the methionine-choline deficient diet (MCD) [37]. Succinate-driven liver respiration was also the most affected in rats fed alcohol [38]. SDH creates the only direct functional link between the tricarboxylic acid cycle (TCA) and OXPHOS, and it is optimally situated to coordinate flux through both pathways. Unlike complex I, the entry of electrons into the coenzyme Q pool from SDH is not constrained by a high proton-motive force [39,40,41]. SDH activity is necessary for gluconeogenesis, and succinate-driven respiration is particularly high in liver mitochondria in comparison with other tissues [39,42]. SDH predominance for driving O_2_ consumption is linked with the capacity to support the highest rate of ROS production in mammalian mitochondria [43]. In vivo, SDH is a relevant producer particularly in reverse electron transfer through complex I or through matrix NAD^+^ dehydrogenases when multiple substrates feed electrons into the coenzyme Q pool, but there is limited energy demand [44,45]. Under these conditions, inhibition of SDH leads to a reduction in ROS generation and decreases substrate flux through the TCA and anaplerotic/cataplerotic pathways [40]. We did not find significant differences among groups in terms of gene and protein expression of the SDHA subunit; however, we cannot exclude changes in SDH expression because we did not monitor other SDH subunits or assembly factors. The catalytic activity of SDH is also modulated by post-translational modifications and active site inhibition by TCA cycle intermediates [41,46]. SDH inhibition could represent an adaptive mechanism to prevent OXPHOS overflow and massive ROS production. However, inactivation of SDH leads to the accumulation of succinate, an important signaling molecule associated with inflammation, fibrosis, and carcinogenesis [41]. Obesity, type 2 diabetes, alcohol-induced liver injury, and NASH were associated with elevated levels of circulating succinate [47,48]. As a regulator of inflammation and fibrosis, succinate activates inflammatory and hepatic stellate cells [37].

We also found an increased ketogenic capacity in mice fed the WD, although the capacity for FA oxidation was not affected. Using octanoylcarnitine as a substrate, we neglected the regulatory role of carnitine palmitoyltransferase I. A unique feature of liver mitochondria is that hepatic TCA is not requisitely linked to β-oxidation because acetyl-CoA in excess of hepatocyte energy demand is shunted to ketogenesis [49]. While playing a minor role in the physiological fed state, ketogenesis may be a considerable factor in the state of metabolic inflexibility and in NAFLD etiology [50]. Ketogenesis could represent an ideal pathway by which to clear excess liver lipids. Levels of ketones have been reported to be increased or decreased in individuals with NAFLD [51,52]. It appears that, during the onset of obesity and hepatosteatosis, ketogenesis is activated, but later diminishes [51]. Impaired ketogenesis induced by an obesogenic diet or/and by genetic manipulation is linked to an increase in TCA flux, gluconeogenesis, oxidative stress, and severe inflammation in both mice and humans [49,50,53]. It was assumed that activation of ketogenesis is a potential therapeutic recourse that may increase lipid disposal [53]. However, chronic hyperketonemia decreases the peripheral oxidation of other substrates, promotes peripheral insulin resistance, and may contribute to whole-body metabolic dysfunction [54].

## 4. Materials and Methods

Animals and diets. All animals received care according to the guidelines set by the Animal-welfare Body of the Charles University (Prague, CZE). The animals were maintained under controlled conditions at 23  ±  1  °C, 55%  ±  10% relative humidity, air exchange 12–14 times/h, 12-h light–dark cycle periods, and free access to food and water. Male C57BL/6J mice (26  ±  2 g, Velaz, Czech Republic) were randomly assigned to two groups (*n* = 6, each group) and fed ad libitum with either a standard control diet (CD, PicoLab RD 20, LabDiet) and tap water or a Western-style diet (WD, AIN-76A WD, TestDiet) and glucose (18.1 g/L), fructose (24 g/L) provided in water over 24 weeks. The mice were sacrificed under anesthesia, and blood, epididymal fat, and liver samples were harvested for further analysis; kept on ice during processing; or immediately frozen in liquid nitrogen and stored at −80 °C.

Blood analysis. Blood analysis was performed using VetScan mammalian liver profile and a VS2 chemical analyzer (Canada).

Histology. Fresh samples of liver and epididymal fat were fixed in 4% neutral formaldehyde, embedded in paraffin, sectioned, and placed on glass slides. Hematoxylin and eosin staining was performed according to standard techniques. Liver fibrosis was quantified using Sirius red staining. The histology was evaluated by a pathologist who was blinded to the dietary condition.

Determination of TGs and cholesterol. Lipids from the livers were prepared using chloroform-methanol extraction [55]. Total cholesterol and TGs were measured using commercial kits (Roche Diagnostics GmbH, Germany). Procedures followed the manufacturer’s protocols.

Preparation of tissue homogenates and supernatants. For the measurement of mitochondrial respiration, ROS production, determination of GSH, and estimation of SDH activity, 10% liver homogenates in cold MIR05 medium (0.5 mM EGTA, 3 mM MgCl_2_, 60 mM K-lactobionate, 20 mM taurine, 10 mM KH_2_PO_4_, 20 mM HEPES, 110 mM sucrose and 1 g/L bovine serum albumin, fatty acid free, pH 7.1) were immediately prepared using a Potter–Elvehjem homogenizer. The homogenates were centrifuged (5 min, 800× *g*, 4 °C), and the lipid layer was removed. Protein content was evaluated by the Bradford method [56]. For determination of liver cytokines, TBARS and Western blot analysis (WB), frozen liver samples (0.2 g for cytokines and TBARS and 0.1 g for WB) were homogenized in 0.9 mL or in 1 mL (for WB) of precooled RIPA buffer, kept on ice for 2 h, and centrifuged twice (10,000× *g*, 4 °C, 10 min). The supernatants were aliquoted and either immediately used for assessment or stored at −80 °C until analysis. Protein content was measured by the bicinchoninic acid method [57].

Determination of liver cytokines. Concentrations of TNF-α and IL-6 in the supernatant were measured by enzyme-linked immuno sorbent assay (BMS622, BMS603, Bender MedSystems, Austria) according to the manufacturer’s instructions.

Western blot analysis. The proteins (100 µg) were applied on Novex NuPAGE 4%–12% Bis-Tris gel (Invitrogen) under nonreducing conditions. The proteins were transferred to a 0.2 mm Hybond nitrocellulose membrane (GE Healthcare). Ponceau S was the stain used, and β-actin was used as a loading control. The membranes were incubated with antibodies to β-actin (AC-74, Sigma), p21 (F-5, Santa Cruz), p53 (BP53-12, Exbio), Bcl-2 (C-2, Santa Cruz), Bax (B-9, Santa Cruz), TIMP-1 (R-18, Santa Cruz), α-SMA (1A4, Sigma), TGF-β1 (V, Santa Cruz), Col1A2 (COL-1, Santa Cruz), and SDHA (B-1, Santa Cruz) at 4 °C overnight. The secondary antibodies were from Santa Cruz. Detection was performed with Western blotting luminol reagent (Santa Cruz). The blots were scanned and quantified using PXi imaging system (Syngene, UK).

RNA isolation and quantitative polymerase chain reaction (qPCR). Total cellular RNA was extracted by TRIzol reagent (Invitrogen) according to the method of Chomczynski and Sacchi [58]. RNA was reverse transcribed using a cDNA reverse transcription kit and quantified with TaqMan Gene Expression Assays (Acta1 Mm00808218_g1, Fndc3a Mm01312526_m1, TGFb1 Mm03024053_m1, col1a2 Mm01165186_m1, TIMP-1 Mm01341361_m1, Bax Mm01205549_m1, Bcl2 Mm00477631_m1, Cdkn1a Mm00432448_m1, Trp53 Mm01731287_m1, SDHA Mm01352357_m1, Ucp2 Mm00627599_m1). Gene expression was analyzed using the QuantStudio 6 real-time PCR system (all obtained from Applied Biosystems, Czech Republic). The results were normalized to the 18S RNA expression.

Markers of hepatic oxidative stress. The lipoperoxidation was determined by the assessment of MDA as TBARS [59]. ROS production was monitored by using 5- (and 6-)chloromethyl-2’,7´-dichlorodihydrofluorescein diacetate (CM-H2DCFDA, Invitrogen). The liver homogenate (0.1 mg/mL) was incubated in potassium medium (100 mM KCl, 10 mM Tris-HCl, 3 mM MgCl_2_, 4 mM KH_2_PO_4_, 1 mM EDTA, pH 7.4) containing 1 μM CM-H2DCFDA and a mixture of respiratory substrates. The substrates used and their concentrations are given in a graph legend (Figure 7). The formation of dichlorofluorescein was measured for 1 h in 5 min intervals in a Tecan Infinite M200 (λ_Ex_ = 485 nm, λ_Em_ = 535 nm, 37 °C). For the determination of GSH, the liver homogenate was added to cold 10% metaphosphoric acid, shaken, and centrifuged (20,000× *g*, 10 min, 4 °C). Glutathione in the supernatant was analyzed by a modified fluorimetric method [60], as described previously [61]. Mitochondrial respiration. Mitochondrial respiration was assessed by high-resolution respirometry (OROBOROS Oxygraph-2k, Austria) using the two substrate-uncoupler-inhibitor-titration (SUIT) protocols (Figure 9). Measurements were performed in MiR05 medium at 37 °C. The terminology we used for the description of respirometric results is broadly explained in a paper in preparation by the COST MitoEAGLE project consortium investigators [62].

Activity of succinate dehydrogenase. In liver homogenate, the activity of SDH was measured specrophotometrically using p-iodonitrotetrazolium as an artificial electron acceptor [63].

Statistical analysis. The data are expressed as the means ± standard deviation (SD). All data processing was performed using GraphPad Prism 6.01 software (La Jolla, CA, USA). Group comparisons were performed using the Mann Whitney U test.

## 5. Conclusions

In conclusion, feeding mice the WD induced massive liver steatosis accompanied by mild inflammation and fibrosis in our experimental model. We found decreased succinate-activated mitochondrial respiration and decreased SDH activity. These findings appear to reflect a general response to chronically increased substrate flux in the mitochondria because diminished succinate-activated respiration and/or SDH activity was also found in the liver of MCD-fed mice [37], alcohol-fed rats [38], and genetically obese db/db mice [36]. Inhibition of SDH could lead to the accumulation of succinate, an important signaling molecule associated with inflammation, fibrosis, and carcinogenesis [39,41]. We also found increased liver ketogenic capacity. It appears that mitochondria in this stage of the disease have adapted to the increased substrate influx. The adaptations are complex and prevent TCA and OXPHOS overflow and massive ROS production. However, the capacity of these adaptations is not limitless, and liver metabolism must be regulated with respect to peripheral tissues.

## Figures and Tables

**Table 1:** Phenotype and blood biochemistry.

Parameter	CD	WD
Body weight, g	30.5 ± 3.27	45.17 ± 4.02 **
Absolute liver weight, g	1.42 ± 0.18	3.58 ± 0.88 **
Relative liver weight, %	4.65 ± 0.46	7.83 ± 1.38 **
Epididymal fat weight, g	0.80 ± 0.25	2.17 ± 0.27 **
ALT, µkat/L	0.45 ± 0.08	5.23 ± 2.38 **
ALP, µkat/L	0.62 ± 0.42	1.50 ± 0.55
Cholesterol, mmol/L	2.52 ± 0.45	6.82 ± 0.75 **
Bile acids, µmol/L	< 1	10.00 ± 3.16 **
Total bilirubin, µmol/L	3.25 ± 0.96	3.75 ± 0.50
BUN, mmol/L	6.55 ± 1.01	4.58 ± 0.83 **
albumin, g/L	26.67 ± 7.17	28.00 ± 2.92
GGT, µkat/L	< 0.1	< 0.1

The results are expressed as the means ± SD; ** *p* < 0.01 (*n* = 6 each group). CD, control diet; WD, Western-style diet.

## Abbreviations

α-SMA	Alpha smooth muscle actin
ADP	Adenosine diphosphate
ALP	Alkaline phosphatase
ALT	Alanine transaminase
AT	Adipose tissue
BUN	Blood urea nitrogen
CD	Control diet
CoA	Coenzyme A
Col1A2	Collagen type I alpha 2 chain
ETS	Electron transfer system
eWAT	Epididymal white adipose tissue
FADH_2_	Reduced flavin adenine dinucleotide
FAs	Fatty acids
GGT	γ-glutamyl transferase
GSH	Reduced glutathione
HFD	High-fat diet
IL-6	Interleukin 6
MCD	Methionine-choline deficient diet
MDA	Malondialdehyde
NADH	Reduced nicotinamide adenine dinucleotide
NAFLD	Nonalcoholic fatty liver disease
NASH	Nonalcoholic steatohepatitis
OXPHOS	Oxidative phosphorylation
p21	Cyclin-dependent kinase inhibitor 1
p53	Tumor protein p53
ROS	Reactive oxygen species
ROX	Residual oxygen consumption
SDH	Succinate dehydrogenase
SDHA	Succinate dehydrogenase subunit A
SUIT	Substrate-uncoupler-inhibitor-titration
TBARS	Thiobarbituric acid reactive substances
TCA	Tricarboxylic acid cycle
TGF-β	Transforming growth factor beta
TGs	Triglycerides
TIMP-1	Tissue inhibitor of metalloproteinases-1
TNF-α	Tumor necrosis factor alpha
UCP-2	Uncoupling protein 2
WD	Western-style diet
